# White matter microstructural perturbations after total sleep deprivation in depression

**DOI:** 10.3389/fpsyt.2023.1195763

**Published:** 2023-06-28

**Authors:** Brandon Taraku, Artemis Zavaliangos-Petropulu, Joana R. Loureiro, Noor B. Al-Sharif, Antoni Kubicki, Shantanu H. Joshi, Roger P. Woods, Randall Espinoza, Katherine L. Narr, Ashish K. Sahib

**Affiliations:** ^1^Department of Neurology, Ahmanson-Lovelace Brain Mapping Center, University of California, Los Angeles, Los Angeles, CA, United States; ^2^Department of Psychiatry and Biobehavioral Sciences, University of California, Los Angeles, Los Angeles, CA, United States

**Keywords:** sleep deprivation, white matter, depression, diffusion imaging, rumination

## Abstract

**Background:**

Total sleep deprivation (TSD) transiently reverses depressive symptoms in a majority of patients with depression. How TSD modulates diffusion tensor imaging (DTI) measures of white matter (WM) microstructure, which may be linked with TSD’s rapid antidepressant effects, remains uncharacterized.

**Methods:**

Patients with depression (*N* = 48, mean age = 33, 26 women) completed diffusion-weighted imaging and Hamilton Depression Rating (HDRS) and rumination scales before and after >24 h of TSD. Healthy controls (HC) (*N* = 53, 23 women) completed the same assessments at baseline, and after receiving TSD in a subset of HCs (*N* = 15). Tract based spatial statistics (TBSS) investigated voxelwise changes in fractional anisotropy (FA) across major WM pathways pre-to-post TSD in patients and HCs and between patients and HCs at baseline. *Post hoc* analyses tested for TSD effects for other diffusion metrics, and the relationships between change in diffusion measures with change in mood and rumination symptoms.

**Results:**

Significant improvements in mood and rumination occurred in patients with depression (both *p* < 0.001), but not in HCs following TSD. Patients showed significant (*p* < 0.05, corrected) decreases in FA values in multiple WM tracts, including the body of the corpus callosum and anterior corona radiata post-TSD. Significant voxel-level changes in FA were not observed in HCs who received TSD (*p* > 0.05). However, differential effects of TSD between HCs and patients were found in the superior corona radiata, frontal WM and the posterior thalamic radiation (*p* < 0.05, corrected). A significant (*p* < 0.05) association between change in FA and axial diffusivity within the right superior corona radiata and improvement in rumination was found post-TSD in patients.

**Conclusion:**

Total sleep deprivation leads to rapid microstructural changes in WM pathways in patients with depression that are distinct from WM changes associated with TSD observed in HCs. WM tracts including the superior corona radiata and posterior thalamic radiation could be potential biomarkers of the rapid therapeutic effects of TSD. Changes in superior corona radiata FA, in particular, may relate to improvements in maladaptive rumination.

## 1. Introduction

Major depression is a common and debilitating disorder that affects 2–6% of people across the globe each year (1). Though mostly treatable, standard antidepressant medications are slow to act, typically taking weeks to months to yield clinical benefits (2). Identifying brain changes that play a role in depression recovery over these protracted time frames can thus be challenging. Lack of sleep can cause cognitive difficulties, perceptual abnormalities and negatively impact mood and anxiety in healthy individuals (3, 4). Paradoxically, one night of total sleep deprivation (TSD) is known to produce antidepressant response within 24 h in ∼60% of subjects with depression (5, 6). Although the therapeutic effects of TSD are only brief and symptoms return after a full night of recovery sleep (7), its rapid effect on mood serves as a model to understand the biological mechanisms associated with fast-acting antidepressant response. Early neuroimaging studies have linked the clinical response of TSD to metabolic changes in the anterior cingulate (ACC) (8, 9), which is a brain region frequently implicated in other depression treatment studies (10). However, there are limited neuroimaging studies (11, 12) that have attempted to identify the neural correlates of TSD-related therapeutic effects in patients with depression.

Diffusion-weighted imaging (DWI) is a neuroimaging technique that can be used to evaluate the diffusion properties of water within brain tissue. Diffusion tensor imaging (DTI), which relies on the tensor model to estimate the direction and magnitude of diffusion, is typically used to study the microstructural integrity of white matter (WM) tracts *in vivo* (13, 14). Measures of fractional anisotropy (FA), and axial (AD), and radial diffusivity (RD), which estimate the directionality of diffusion anisotropy, and diffusion along the principle or perpendicular axes of diffusion, respectively, are frequently evaluated with DTI. Mean diffusivity (MD), which represents the magnitude of diffusion independent of direction (15, 16) is also commonly measured. Many previous DTI studies have found abnormalities in WM microstructure in patients with depression, typically reporting decreased FA in tracts within frontal, occipital and parietal WM, and the corpus callosum (17–21). Reduced WM FA is shown to associate with the recurrence or the severity of depression (19, 22). Further, antidepressant response is shown to associate with changes in FA or other diffusion metrics (23–26), including for rapidly acting treatments such as electroconvulsive (ECT) and ketamine therapy (26, 27). Understanding how TSD affects the functional and structural integrity of neural pathways associated with depressive symptoms could provide valuable insights with regard to its rapid antidepressant action and potentially inform the development of other effective fast-acting antidepressant interventions. However, to the best of our knowledge the effect of TSD on WM microstructure in patients with depression is yet uncharacterized.

To better understand the neural processes contributing to the rapid-acting antidepressant action of sleep deprivation, the current study sought to investigate whether TSD elicits changes in cerebral WM microstructure in patients with depression. Since the lack of prior data warrants investigation of all major WM pathways, we used tract-based spatial statistics (TBSS) analysis to examine changes diffusion properties at the voxel-level. However, based on previous findings of TSD in controls (28, 29), we hypothesized TSD would result in change in WM microstructure and this change would be significantly different between controls and patients with depression who received TSD. Furthermore, as rumination is known to have a mediatory effect between mood and sleep deprivation (30), we hypothesized that WM regions associated with TSD response would also relate to changes in rumination.

## 2. Materials and methods

### 2.1. Participants

Participants included 48 individuals with depression evaluated using the Structured Clinical Interview for DSM-5-Research Version [SCID-5-RV, (31)] and 53 healthy controls (HC). Subjects were recruited from the Los Angeles area through advertisements and clinician referral. All patients, and a subset of HCs (*N* = 15) participated in a sleep deprivation session of at least 24 h under controlled conditions at the UCLA Clinical and Translational Research Center. Clinical assessments and MRI brain imaging data was acquired the day before TSD (TP1) and immediately after TSD (TP2). At each time point, depression severity was assessed using the Hamilton Depression Rating Scale (HDRS), 17–item (HDRS sleep scores were adjusted to include the scores of sleep 1-week prior to the study) (32).

Exclusion criteria for all participants included any unstable medical or neurological condition, current substance abuse or dependence (ascertained by laboratory testing) or substance abuse history within the preceding 3-months, current or past history of psychosis, schizophrenia, intellectual disability or other developmental disorder, diagnosis of dementia and any contraindication to scanning (e.g., metal implants or claustrophobia). Prior to treatment, all patients had mild to severe depressive symptoms (HDRS score > = 14) (32). All subjects provided written informed consent following procedures approved by the University of California, Los Angeles (UCLA) Institutional Review Board (IRB).

### 2.2. TSD procedure

Total sleep deprivation occurred in a private room at the UCLA Clinical and Translational Research Center. During the overnight TSD session, participants were continuously monitored to ensure wakefulness. Subjects were provided regulated meals and snacks, allowed to read, watch movies, use the internet, talk to staff, and walk around the clinic. Subjects were not allowed to lay down or turn off the lights and were restricted from consuming caffeine. Nursing staff frequently checked to ensure that subjects did not fall asleep or appear to be drowsy. MRI scans and clinical/behavioral assessments took place before and directly after the 24 h overnight sleep deprivation session. Pre and post TSD scans and assessments were collected in the late afternoon for all participants. Since subjects were not permitted to sleep on the day of baseline scans, all had experienced at least 36 h of continuous wakefulness by the time of the post-TSD assessments.

### 2.3. MRI acquisition

Imaging data was acquired using a Siemens 3T Prisma MRI system at UCLA’s Brain Mapping Center and a 32-channel phased array head coil. Image acquisition sequences were identical to those used by the Human Connectome Project (HCP) Lifespan studies for Aging and Development (33). Scans consisted of a T1-weighed (T1w) multi-echo MPRAGE with voxel size (VS) = 0.8 mm isotropic; repetition time (TR) = 2,500 ms; echo time (TE) = 1.81:1.79:7.18 ms; inversion time (TI) = 1,000 ms; flip angle (34) = 8.0*^o^*; and acquisition time (TA) = 8:22 min, and a T2-weighted (T2w) acquisition with VS = 0.8 mm isotropic; TR = 3,200 ms; TE = 564 ms; and TA = 6:35 min, both with real-time motion correction (34). Diffusion data were collected using a multiband (MB), echo-planar imaging sequence with 1.5 mm isotropic spatial resolution. Four consecutive diffusion MRI runs were collected with anterior-posterior (AP) (2 runs) and posterior-anterior (PA) (2 runs) phase encoding polarities. Each run contained interleaved shells with two diffusion weightings (*b* = 1,500 and 3,000 s/mm^2^), comprising 185 diffusion directions in total across both scans within each phase encoding direction (33).

### 2.4. DTI preprocessing

Anatomical and diffusion data were visually inspected and minimally preprocessed using the HCP minimal preprocessing pipeline (35–37) implemented using the BIDS-App (38). The HCP Diffusion pipeline included intensity normalization across runs, the “TOPUP” algorithm for EPI distortion correction, the “EDDY” algorithm for eddy current and motion correction, gradient non-linearity correction, registration of mean b0 images to native T1w space with FLIRT BBR and transformation of diffusion data, gradient deviation, and gradient directions to 1.5 mm structural space. Following eddy correction (39), the diffusion scans were again examined visually to screen for potential artifacts. Automated eddy QC tools (40) were subsequently applied to quantify the extent of head motion within and across study participants (average motion: 1.14 mm, 0.056 SD: average relative motion: 0.46 mm, 0.11 SD). No subjects were excluded for motion based on eddy QC outputs. Diffusion tensor models were then fitted independently for each voxel within the brain mask (based on FreeSurfer segmentation) and images of FA, first (λ1), second (λ2), and third (λ3) eigenvalues were generated for each participant using the FSL-FDT toolkit (41).

Using the TBSS tool (42), FA images from each participant were then aligned to the FMRIB58_FA template and transformed into Montreal Neurological Institute 152 1 mm^3^ standard space. Next, an average FA image was generated and thinned to create a WM skeleton representing the centers of all WM tracts common to all participants. This FA skeleton was then thresholded to FA ≥0.2 to include all major WM pathways. Each participant’s aligned FA image was projected onto the mean FA skeleton. Voxel-wise statistics were subsequently performed on this skeletonized participant data using the FSL randomize tool (43).

### 2.5. Statistical analysis

Voxel-level statistical analysis focused on addressing the following comparisons: (1) Effects of time for patients with depression pre to post TSD using a paired *t*-test, (2) Effects of time for HCs pre to post TSD using a paired *t*-test, and (3) Differential effects of time (or the interaction) by comparing change in FA (ΔFA) following TSD between patients with depression and HCs, including age and sex as regressors of no interest. For all comparisons, permutation testing included 5,000 permutations with the threshold-free cluster enhancement (TFCE) option (44). Statistical maps were corrected for multiple comparisons (family wise error, FWE *P* < 0.05). The Johns Hopkins University International Consortium for Brain Mapping (JHU ICBM)-DTI-81 WM labels atlas (45) determined the WM pathways showing significant FA differences. For clusters not labeled using this atlas, assignments were made using the MNI structural template in FSL.

Several *post hoc* tract-wise regions of interest (ROI) analyses were subsequently performed. These included establishing whether (1) changes in FA in regions showing significant effects of TSD in patients, differed between patients and all controls at baseline, (2) effects of TSD were present for other diffusion metrics (AD, RD, and MD) in regions showing FA changes, and finally if (3) changes in extracted FA, AD, RD, and MD values associated with % change [(TP1–TP2)/TP1] in mood or rumination scores. For *post hoc* analyses, a binary mask of each significant cluster identified in the whole brain voxel-wise tract analysis was generated to extract the mean FA within the cluster for each participant. The cluster binary mask was similarly used to calculate AD (λ1 values), RD (average of the λ2 and λ3 values), and MD (average diffusion) after the same non-linear warping parameters were used to project the non-FA images onto the TBSS WM skeleton. These tests were conducted using IBM Statistical Packages for the Social Sciences (SPSS v26). A *p*-value of < 0.05 was used to establish statistical significance for *post hoc* analyses.

## 3. Results

### 3.1. Demographic and clinical results

**FIGURE 1:**
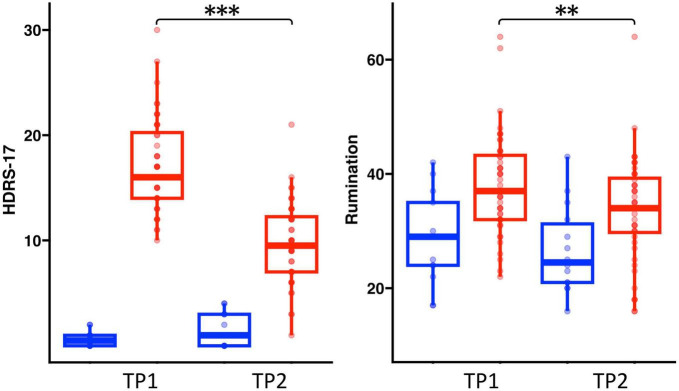
Box plots showing the distribution of HDRS and rumination scores in healthy controls (Blue) and patients (Red) pre-to-post TSD (^**^p < 0.01 and ^***^p < 0.001).

Age and sex did not significantly differ between HC and patients with depression (Table 1). There was a significant diagnostic group by time interaction for HDRS [*F*(1,58) = 23.13, *p* < 0.001] and a trend of an interaction for rumination scores [*F*(1,58) = 3.47, *p* = 0.06]. In patients, HDRS [*t*(47), *p* < 0.0001] and rumination [*t*(1, 47), *p* < 0.0009] scores showed significant improvement after TSD. Rumination scores did not change significantly for HC following TSD, though HDRS scores significantly increased (worsened) [*t*(14) = 3.02, *p* = 0.01] (Figure 1).

**TABLE 1:** Demographic and clinical information for patients and controls.

	HC Mean (SD)	MDD Mean (SD)	T/χ-value	*P*-value
Number of subjects (*N*)	53	48		
Gender (% female)	51.85	55.1	*χ* = 0.06	0.8
Age (years)	32.14 (12.1)	33.20 (10.5)	T = 0.24	0.81
Duration lifetime illness (years)		17.52 (15.66)		
Current episode (years)		2.15 (4.07)		
*Comorbid disorders				
Generalized Anxiety		21		
Manic episodes		1		
Feeding and Eating Disorders		6		
Trauma and stressor related disorders		11		
HDRS (TP1)	1.01 (1.6)	17.06 (4.3)		
HDRS (TP2)	1.73 (1.53)	13.47 (4.3)		
RRS (TP1)	27.22 (9.4)	38.06 (8.7)		
RRS (TP2)	27.08 (7.6)	33.60 (9.1)		

HDRS, Hamilton Depression Rating Scale; RRS, ruminative response styles; HC, healthy controls; patients with major depressive disorder (MDD) at baseline (TP1); 24 h after TSD (TP2).

*Comorbid disorders based on SCID -V.

### 3.2. FA changes after TSD

**FIGURE 2:**
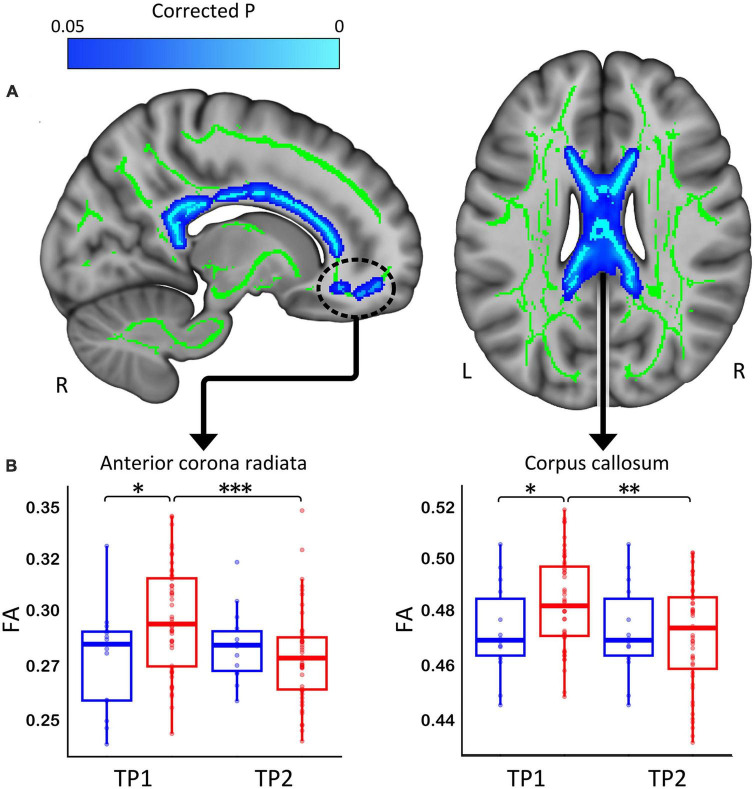
**(A)** White matter clusters showing significant decrease (in blue, *p* < 0.05 FWE corrected) in fractional anisotropy (FA) in the corpus callosum and the anterior corona radiata. **(B)** Box plots showing the distribution of FA values for healthy controls (Blue) and patients (Red) for the corpus callosum and anterior corona radiata (*p < 0.05, ^**^p < 0.01, and ^***^p < 0.001). R, right, L, left.

Patients with depression showed significant decreases in FA following TSD in multiple WM tracts (*p* < 0.05 FWE correction), including in the body of corpus callosum and the anterior corona radiata (Figure 2A). The paired *t*-test comparing baseline (TP1) and post TSD (TP2) for HCs did not reveal significant FA changes at the voxel level (*p* > 0.05 FEW) (null results not shown).

### 3.3. Cross-sectional effects of FA between diagnostic groups at baseline

*Post hoc* analysis revealed that WM regions showing a significant decrease in FA following TSD in patients differed between patients and HCs at baseline (*p* < 0.05) (Figure 2B shows these effects graphically and Table 2 provides statistical details for significant follow-up comparisons).

**TABLE 2:** Statistical descriptors for clusters showing significant voxel-level effects.

Cluster	#voxels and signal peaks (*x,y,z*)	MDD T1 Mean ± SD	MDD T2 Mean ± SD	*T*-value	*P*-value
**FA changes after TSD in patients**					
Corpus Callosum	5,009 (86, 103, 96)	0.481 ± 0.02	0.466 ± 0.03	−3.58	8.1E-04
Anterior corona radiata	135 (76, 178, 62)	0.297 ± 0.03	0.280 ± 0.02	−4.05	1.8E-04
Posterior Thalamic Radiation	37 (54, 72, 68)	0.390 ± 0.03	0.371 ± 0.04	−3.12	3.1E-03
**Cluster**	**#voxels and signal peaks (*x,y,z*)**	**HC T1** **Mean ± SD**	**HC T2** **Mean ± SD**	***T*-value**	***P*-value**
**FA changes after TSD in controls**					
Superior corona Radiata	69 (69, 102, 115)	0.349 ± 0.03	0.383 ± 0.02	5.02	2.4E-04
Frontal Lobe	34 (62, 155, 67)	0.244 ± 0.02	0.297 ± 0.05	5.15	1.9E-04
**Cluster**	**#voxels and signal peaks (*x,y,z*)**	**MDD T1** **Mean ± SD**	**HC T2** **Mean ± SD**	***T*-value**	***P*-value**
**Cross-sectional effects of FA between diagnostic groups at baseline**					
Anterior corona Radiata	135 (76, 178, 62)	0.390 ± 0.03	0.362 ± 0.02	2.46	1.6E-02
Corpus Callosum	5,009 (86, 103, 96)	0.481 ± 0.02	0.467 ± 0.02	2.05	4.5E-02
**Cluster**	**#voxels and signal peaks (*x,y,z*)**	**MDD T1** **Mean ± SD**	**MDD T2** **Mean ± SD**	***T*-value**	***P*-value**
**RD changes after TSD in patients**					
Posterior Thalamic Radiation	34 (62, 155, 67)	6.78E-04 ± 3.9E-05	6.96E-04 ± 4.1E-05	2.42	0.02
**Cluster**	**#voxels and signal peaks (*x,y,z*)**	**HC T1** **Mean ± SD**	**HC T2** **Mean ± SD**	***T*-value**	***P*-value**
**RD changes after TSD in patients**					
Frontal Lobe	37 (54, 72, 68)	7.28E-04 ± 4.1E-05	6.80E-04 ± 3.1E-05	−4.43	6.78E-04

### 3.4. Differential TSD effects of FA between patients and HCs

**FIGURE 3:**
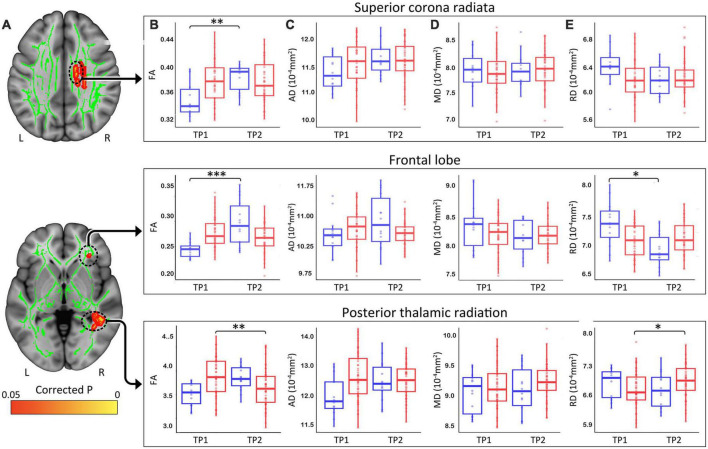
**(A)** White matter clusters showing significant difference (orange, *p* < 0.05 FWE corrected) for change in fractional anisotropy (FA) pre-to-post TSD between controls and patients. **(B)** Box plots showing distribution of FA, axial **(C)**, radial **(D)**, and mean **(E)** diffusivity values for healthy controls (Blue) and patients (Red) in the superior corona radiata, frontal lobe and the posterior thalamic radiation pre and post TSD. (*p < 0.05, ^**^p < 0.01, and ^***^p < 0.001). R, right, L, left.

At *p* < 0.05 FWE correction and including age and sex as covariates of no interest, the two-sample *t*-test comparing ΔFA (TP1–TP2) between HCs and patients with depression at the voxel level revealed multiple significant WM clusters, including the superior corona radiata, a cluster in frontal lobe WM and the posterior thalamic radiation (Figure 3A).

These clusters were used to create ROIs to extract regional FA (Figure 3B), AD (Figure 3C), MD (Figure 3D), and RD (Figure 3E) values for HC and patients. Post TSD, HCs showed a significant increase of mean FA values in the superior corona radiata and a WM cluster in the frontal lobe, while patients showed a significant decrease of mean FA value in the posterior thalamic radiation (Figure 3B). Among the other DTI measures, only RD showed a significant decrease in frontal lobe WM for HCs post TSD, while patients showed a significant increase in RD values in the posterior thalamic radiation (Figure 3E). Table 2 provides statistical details for regional effects observed as significant.

### 3.5. Associations with mood and rumination in patients

**FIGURE 4:**
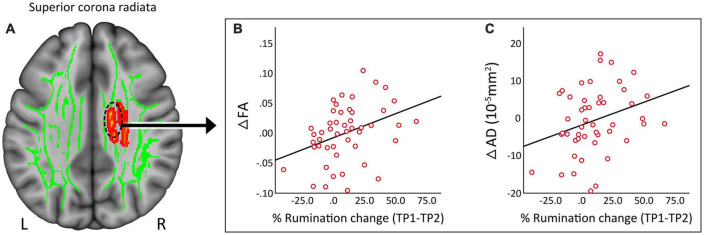
**(A)** Regional correlates of DTI measures from the superior corona radiata. **(B)** Change in fractional anisotropy (FA) showed a significant (*p* = 0.01) positive correlation with change in rumination. **(C)** Change in axial diffusivity (AD) showed a significant (*p* = 0.038) positive correlation with change in rumination. R, right, L, left.

Clusters that survived statistical significance in the whole brain voxel-level tract analysis (ΔFA, *p* < 0.05 FWE TFCE) were used to create ROIs of average FA, AD, MD, and RD values. None of the DTI measures showed any significant relationship with change in HDRS scores post TSD in patients. However, the change in the superior corona radiata (Figure 4A) for FA (Figure 4B, *r* = 0.364, *p* = 0.011) and AD (Figure 4C, *r* = 0.301, *p* = 0.038) showed a significant correlation with % change in rumination scores.

## 4. Discussion

Total sleep deprivation is widely reported to produce rapid onset antidepressant effects within 24–48 h in at least half of depressed patients who receive it (12, 46). Although symptoms return immediately after recovery sleep (6), TSD presents an important opportunity to understand the mechanisms contributing to rapid antidepressant response. How TSD modulates structural networks to influence antidepressant response remains unstudied. By leveraging *in vivo* diffusion imaging methods, the current study thus sought to investigate whether TSD affects WM microstructure in patients with depression and in controls, and to establish whether observed changes in WM associate with improvements in mood and rumination. Primary findings from this investigation showed that TSD promotes significant reductions of FA within major WM pathways, including the body of corpus callosum and anterior corona radiata in patients with depression. Further, significant interactions of TSD-related change between patients and controls were present for FA (ΔFA) in numerous WM tracts, including the superior corona radiata, frontal WM and the posterior thalamic radiation. Here, patients and HCs mostly exhibited opposite directions of mean change in particular WM pathways. Finally, changes in FA and AD within the superior corona radiata showed a significant positive relationship between improvement in rumination pre-to-post TSD. These findings suggest that while some of observed regional reductions in FA may relate to the vulnerability of insufficient sleep (29), TSD-related changes in WM microstructure positively impact ruminative thought focused on depressive symptoms (47). To the best of our knowledge, this is the first report addressing the immediate effects of TSD on WM microstructure and its behavioral correlates in patients with depression.

### 4.1. WM microstructural changes after TSD

The current study chose to focus on FA for primary analyses since it reflects the directional selectivity of diffusion and is sensitive to both direction and amount of hindrance/restriction and direction of water molecules (48, 49). We observed significant widespread reduction of FA values in multiple WM tracts in patients with depression post TSD. Regions of FA decreases mainly included the corpus callosum and tracts traversing the frontal WM. Tract-based estimates of FA are shown to be highly reproducible across time when the DWI sequence, scanner software and system remain stable as was the case for DWI acquisition in this study (39, 50, 51). Significant changes in FA observed following TSD in patients (∼4% mean decrease across significant clusters) were of slightly larger magnitude to those reported previously in healthy subjects following 24 h of sleep deprivation (2–3% mean decrease across significant clusters) (28). Decreases in FA observed in a prior study comparing normal sleep-wake cycles to 24–32 h of sleep deprivation in healthy subjects were also similar, though only extra-neurite mean diffusivity (exMD), which is highly correlated with FA, was significant (52).

Though not fully understood at the molecular and cellular level, the antidepressant mechanisms of TSD are suggested to involve the modulation neurotrophic and inflammatory processes and changes in endocrine function (4, 53). TSD’s antidepressant effects are suggested as attributable to some of these same factors, including modulation of brain-derived neurotrophic factor (BDNF), vascular endothelial growth factor (VEGF), serotonin, cortisol, and tumor necrosis factor-alpha (TNF-α) (54). Some prior evidence suggests that sleep deprivation in healthy populations increases reactivity in mesolimbic reward brain networks in response to positive as well as negative stimuli (55) as well as decreases connectivity between the ACC and reward networks (56), which may contribute to its effects on depression. As potentially relevant to the current findings, previous studies have reported reduced metabolism in frontal regions after sleep deprivation (57, 58) suggesting that frontal executive centers are particularly susceptible to sleep deprivation. Moreover, functional magnetic resonance imaging (fMRI) research has found an overall decrease in regional and global neural activation following sleep deprivation (55, 59). In accordance, sleep deprivation is reported to negatively affect cognitive function, attention, working memory, and psychomotor vigilance (60–62). Though a prior investigation in controls has reported significant decreases in FA following sleep deprivation (28), in the current study reduced FA at the voxel-level was only observed as significant following TSD in patients. Still, these results in the context of limited prior reports may suggest that the decrease in FA observed across commissural and frontal association pathways are an indication of the sleep deprived brain. However, results also suggest that these changes associated with behavioral state after loss of sleep simultaneously act to relieve depressive symptoms. Since FA represents only the direction and magnitude of diffusion in the microenvironment in which water molecules in the WM exist, the exact mechanisms responsible for changes in diffusion signal can only be inferred (48, 49). However, these changes could point to alterations in myelin and axonal microstructure as well as neural swelling associated with brain activity or edema (49, 63), or other biological processes such as glymphatic activity occurring during sleep (64).

### 4.2. Differential effects of TSD between HC and patients

In line with prior results (7), total sleep deprivation was shown to provide immediate relief of mood symptoms in >44% of patients with depression whereas in HCs mood symptoms tended to increase (see Figure 1). Using independent sample *t*-tests, we sought to directly compare differences in FA pre-to-post TSD between patients and controls in whole brain voxel-level tract analysis. On comparing the ΔFA maps between HC and patients post TSD, we identified several WM tracts including the superior corona radiata, frontal WM and the posterior thalamic radiation where FA values were modulated in opposite directions in patients relative to HCs. In *post hoc* analyses, we also evaluated change in AD, MD, and RD, which may reveal further information regarding the influence of TSD on WM microstructural properties. Despite limitations in extrapolating the biological cause of differences in DTI metrics (48, 49), AD has been considered a marker of axonal microstructure, though both increases and decreases may indicate pathology (65–67). In contrast, RD is often considered a marker of myelin though can also signify changes in axonal diameter or density (65–67). Although only RD showed a significant change post TSD for the selected ROIs, as for FA, the diffusivity measures of AD, mean MD and RD were modulated in opposite directions in patients and HCs post TSD. Differences in the modulation of WM microstructure observed between patients with depression and HCs following TSD may suggest that TSDs underlying therapeutic mechanisms are selectively targeted toward differential WM microstructure in patients. Notably, the WM tracts showing changes in FA with TSD in patients with depression have also been implicated in prior cross-sectional studies comparing patients with depression and controls using whole-brain DTI analysis (18–20, 68–70). Some prior evidence further suggests that medication treatments for depression alter or reverse abnormalities in FA. For example, Bracht et al. (71) showed that FA increased in younger patients, but decreased in older patients in the cingulum following successful recovery from depression after standard antidepressant therapy. Ketamine therapy in major depression is also shown to reduce neurite density (correlated with FA) in several WM tracts, including the posterior thalamic radiation, forceps minor (frontal callosal projections) and occipitotemporal pathways (27).

### 4.3. DTI changes associated with symptoms after TSD

Significant improvements in HDRS and rumination scores were observed post TSD in depressed patients. In *post hoc* analysis, we also observed a significant positive correlation between change in FA and AD in the superior corona radiata/body of corpus callosum with change in rumination scores. At least one prior study has shown associations between altered FA in WM tracts connecting frontal, parietal and limbic regions and rumination (72). Further, since the superior corona radiata plays an important role in integrating higher-level cognitive, and perceptual processing (18), WM alterations might contribute to the impairments in memory, executive functioning, and emotional regulation reported in patients with depression (73). The significant associations observed between improvement in ruminative state and FA and AD measures suggest that temporary symptom recovery due to TSD is at least partly attributable to the modulation of WM microstructure.

### 4.4. Limitations

Several limitations need to be acknowledged with regard to the current study. First, only a subset of the HC sample (*N* = 15) underwent TSD, which led to reduced statistical power for identifying WM microstructural changes perturbed by TSD in the HC group specifically. However, the focus of the current study was to investigate the WM perturbations in patients with depression following TSD and associations with change in mood and rumination. Further, as more impervious to unequal samples we compared the magnitude of change in FA across diagnostic groups and observed changes in diffusion metrics tended to change in opposite directions in patients and HCs. In the current study, patient participants were allowed to continue their current antidepressant medications, which though remaining stable at least 6-weeks prior to TSD invention, may have impacted results.

## 5. Conclusion

The present study demonstrates that 24 h of sleep deprivation leads to significant decreases in FA in the corpus callosum and the anterior corona radiata in patients with depression. In line with previous findings, these alterations in WM microstructure may be primarily due to lack of sleep. However, changes in FA post TSD observed in certain tracts such as the superior corona radiata, frontal WM and the posterior thalamic radiation differed in patients and controls. WM alterations in these tracts thus appear specific to depression and may be linked with cognitive and emotional deficits as previously suggested. The association between improvement in rumination and change in FA and AD post TSD further indicate that temporary symptom recovery following TSD relates to changes in WM structure. Future studies may address whether changes in diffusion properties within these fiber pathways relate to improvements in mood and rumination following other fast-acting antidepressant therapies.

## Data availability statement

Unprocessed and minimally preprocessed imaging data and behavioral data used in this publication are available in the PDC 1.0. Release accessible through the NIMH Data Archive (NDA, https://nda.nih.gov/study.html?id=2010).

## Ethics statement

The studies involving human participants were reviewed and approved by The Office of the Human Research Protection Program (OHRPP) at the University of California, Los Angeles. The patients/participants provided their written informed consent to participate in this study.

## Author contributions

AK, KN, JL, AS, SJ, RW, and RE contributed to the conception and design of the study. AK, BT, and AZ-P performed the statistical analysis. AK wrote the first draft of the manuscript. BT wrote the sections of the manuscript. All authors contributed to manuscript revision, read, and approved the submitted version.
